# Supplementary material for: Regression of nonalcoholic fatty liver disease is associated with reduced risk of incident diabetes: A longitudinal cohort study
Source: PLoS One. 2023 Jul 18;18(7):e0288820. doi: 10.1371/journal.pone.0288820 (PMC10353790; doi:10.1371/journal.pone.0288820)
Supplement: Attachment — Submitted filename: Sinn et al.docx [file pone.0288820.s001.docx]

Sinn et al. conducted a study on “Regression of nonalcoholic fatty liver disease is associated with reduced risk of incident diabetes: A longitudinal cohort study”, in which they investigated the association between regression of nonalcoholic fatty liver disease (NAFLD) and the risk of incident diabetes using a large cohort. The retrospective cohort study included 11,260 participants who underwent at least two health check examinations with abdominal ultrasound between 2001 and 2016 at the Samsung Medical Center. NAFLD regression was defined as disappearance of fatty liver on abdominal US imaging at baseline. Diabetes was defined as a fasting serum glucose ≥ 126 mg/dL, HbA1c ≥ 6.5%, a self-reported history of diabetes, or self-reported use of insulin or antidiabetic medications. The study found that NAFLD regression was associated with a lower risk of incident diabetes.

From my perspective, the inclusion of the following revisions could enhance this study:
**Abstract**:

- Include the duration of the study in the abstract, which is important for readers to understand the scope of the study.
- In the first sentence of the abstract, "whether improvement of NAFLD leads to clinical benefit remains uncertain" should be corrected to "whether improvement of NAFLD leads to clinical **benefits** remains uncertain." (Page 4, Line 60)
- Also in methods section, "A cohort of 11,260 adults who had NAFLD in an initial exam" should be corrected to "A cohort of 11,260 adults who had NAFLD **at** an initial exam." (Page 4, Line 64)
- In the results section, "The fully adjusted hazard ratio (HR) for incident diabetes in participants with regressed NAFLD compared to those with persistent NAFLD were 0.81" should be corrected to "The fully adjusted hazard ratio (HR) for incident diabetes in participants with regressed NAFLD compared to those with persistent NAFLD **was** 0.81." (Page 4, Line 72)
- In the results section, "When assessed by NAFLD severity, among participants with low NAFLD fibrosis score (NFS) (< -1.455), participants with regressed NAFLD had a lower risk of incident diabetes than those with persistent NAFLD" should be corrected to "When assessed by NAFLD severity, among participants with **a** low NAFLD fibrosis score (NFS) (< -1.455), participants with regressed NAFLD had a lower risk of incident diabetes than those with persistent NAFLD." (Page 4, Line 73)
- In the results section, "However, in participants with intermediate to high NFS (≥ -1.455), the risk of incident diabetes was not different between NAFLD regression and persistence group" should be corrected to "However, in participants with **an** intermediate to high NFS (≥ -1.455), the risk of incident diabetes was not different between the NAFLD regression and persistence **groups**." (Page 4, Line 76,77)
- In the conclusions section, "This suggests that early intervention for NAFLD, before advanced fibrosis is present, may maximize metabolic benefit from NAFLD regression" should be corrected to "This suggests that early intervention for NAFLD, before advanced fibrosis is present, may maximize **the** metabolic benefit from NAFLD regression." (Page 5, Line 82)

**Introduction**:

- The first sentence in the introduction should be revised to make it clearer. For example, "Nonalcoholic fatty liver disease (NAFLD) is a condition in which the liver accumulates fat without significant alcohol intake, viral hepatitis, medications that would cause fatty liver, or other obvious causes." (Page 6, Line 84)
- In the introduction section, the authors should provide more information on the prevalence of NAFLD and its impact on public health.
- The research question is not clear, and it is not stated explicitly in the introduction section. The authors should state the research question clearly, for example: “Is regression of NAFLD associated with a decreased risk of incident diabetes?”

**Methods**:

- The number of excluded participants in both the text and Figure 1 does not add up to 11,260. (Page 7, Line 117)
- The statistical methods used for the analysis should be described in more detail. The authors should provide more information on how they conducted the analysis, including the statistical tests used and how they controlled for potential confounding factors.
- In the methods section, the authors should describe the characteristics of the study population in more detail, including their age, sex, and other relevant demographic information.
- In the methods section, the authors should provide more information on the follow-up period and the length of time between the second abdominal US exam and the diagnosis of diabetes.
- In the methods section, the authors should describe how missing data were handled, particularly for variables such as alcohol intake.
- The description of how the abdominal ultrasound imaging was performed could be clearer. The type of equipment used for imaging is listed, but the imaging protocols and criteria used to diagnose NAFLD should also be explained.

**Results**:

- In the results section, the authors should present the results in a more organized and clear manner. It is not clear how many participants had regression of NAFLD, and how many developed incident diabetes.

**Discussion**:

- The authors could provide more context for their findings by discussing how their results relate to previous studies on the association between NAFLD regression and incident diabetes. Additionally, the authors should discuss the potential limitations of the study, such as the use of abdominal ultrasound to diagnose NAFLD, which may not be as accurate as liver biopsy.

**Conclusion**:

- In the discussion section, the authors should provide more information on the implications of the study findings and how they relate to previous research in this area.
